# Diversity of interneurons in the lateral and basal amygdala

**DOI:** 10.1038/s41539-020-0071-z

**Published:** 2020-08-03

**Authors:** Jai S. Polepalli, Helen Gooch, Pankaj Sah

**Affiliations:** 1 Queensland Brain Institute, University of Queensland, St Lucia, QLD 4072 Australia; 2 Department of Anatomy, Yong Yoo Lin School of Medicine, National University of Singapore, Singapore, 117594 Singapore; 3 Brain Research Centre and Department of Biology, Southern University of Science and Technology, Nanshan District, Shenzhen, Guangdong Province P.R. China

**Keywords:** Cellular neuroscience, Learning and memory

## Abstract

The basolateral amygdala (BLA) is a temporal lobe structure that contributes to a host of behaviors. In particular, it is a central player in learning about aversive events and thus assigning emotional valence to sensory events. It is a cortical-like structure and contains glutamatergic pyramidal neurons and GABAergic interneurons. It is divided into the lateral (LA) and basal (BA) nuclei that have distinct cell types and connections. Interneurons in the BLA are a heterogenous population, some of which have been implicated in specific functional roles. Here we use optogenetics and slice electrophysiology to investigate the innervation, postsynaptic receptor stoichiometry, and plasticity of excitatory inputs onto interneurons within the BLA. Interneurons were divided into six groups based on their discharge properties, each of which received input from the auditory thalamus (AT) and auditory cortex (AC). Auditory innervation was concentrated in the LA, and optogenetic stimulation evoked robust synaptic responses in nearly all interneurons, drove many cells to threshold, and evoked disynaptic inhibition in most interneurons. Auditory input to the BA was sparse, innervated fewer interneurons, and evoked smaller synaptic responses. Biophysically, the subunit composition and distribution of AMPAR and NMDAR also differed between the two nuclei, with fewer BA IN expressing calcium permeable AMPAR, and a higher proportion expressing GluN2B-containing NMDAR. Finally, unlike LA interneurons, LTP could not be induced in the BA. These findings show that interneurons in the LA and BA are physiologically distinct populations and suggest they may have differing roles during associative learning.

## Introduction

Learning to identify and respond to threats in the environment is essential for survival and conserved across species. In mammals, the formation and retention of these associative memories engages a distributed network of brain regions, with the basolateral amygdala (BLA) playing a central role^1–3^. This form of learning is widely studied using auditory fear conditioning, in which subjects learn to associate a neutral auditory tone (the conditioned stimulus, CS) with an aversive foot shock (the unconditioned stimulus, US). As a result of their repeated temporal association, subjects exhibit defensive responses to the CS alone (the conditioned response, CR), which is driven by outputs from the amygdala in response to the auditory CS information entering the BLA^4–6^.

Much research has focused on the neurobiological properties of synapses carrying auditory information to the amygdala, the regions they innervate, and the changes they undergo following auditory fear conditioning. Broadly speaking, the BLA receives two streams of auditory innervation from the auditory thalamus (AT) and auditory cortex (AC). The current model of fear conditioning suggests that the integration of CS and US information occurs at glutamatergic principal neurons in the BLA and leads to long-term potentiation (LTP) at synapses carrying the auditory CS^7–9^. This amygdala-dependent learning is tightly controlled by synaptic inhibition mediated by local GABAergic interneurons^10–13^.

As in most cortical and hippocampal regions^14,15^, BLA interneurons have been separated into several subtypes, primarily based on expression of cytosolic markers and local connectivity^16,17^. Of these, the two main families are those expressing parvalbumin (PV) and those expressing somatostatin (SOM), and while both are involved in fear learning^10,13^, they are thought to play distinct roles^10^. Although interneuron classification into groups based on cytosolic marker expression has been useful, it is clear that there is large diversity of form and function within each group^14,15,18^. Further, the BLA is histologically divided into the lateral (LA) and basal amygdala (BA), and while these two regions contain different types of principal neurons with distinct connectivity patterns^19^, interneuron populations in the LA and BA are treated as functionally homogenous.

In this study, we aimed to map interneurons in the LA and BA using electrophysiological recordings, comparing auditory innervation and the biophysical properties of their glutamatergic synapses. We show that electrophysiologically, at least six types of interneurons are present in the BLA. Interneurons in the LA and BA receive different auditory input, and their glutamatergic synapses have different properties and distributions. Moreover, LTP was restricted to cortical synapses on LA interneurons, while inputs to interneurons in the BA do not show LTP. These results suggest that interneuron populations within the LA and BA are differentially recruited during auditory fear conditioning, and thus perform distinct roles during associative learning.

## Results

### Six electrophysiological classes of GABAergic neurons in the BLA

**Fig. 1:**
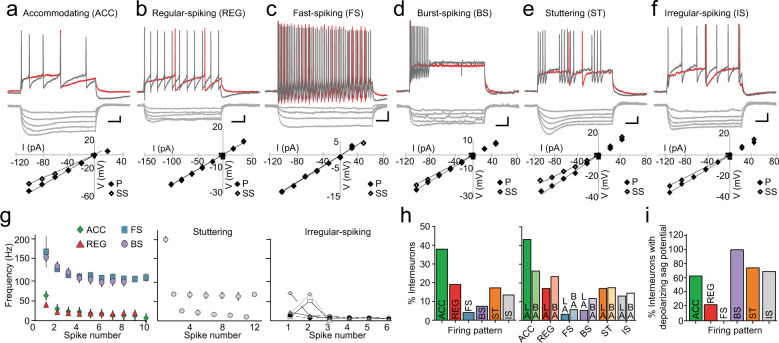
Classification of BLA interneurons based on their intrinsic electrical properties. **a**–**f** Active membrane properties of six distinct BLA interneuron subtypes. *Top*: representative traces of response to threshold (red) and twice-threshold current injection. *Middle*: hyperpolarizing membrane deflection in response to hyperpolarizing current injection. *Bottom*: representative input-output responses for steady state (SS, open diamonds) and peak (P, filled diamonds) voltage responses to hyperpolarizing current injections. (**a**) accommodating cell (ACC), (**b**) regular-spiking cell (REG), (**c**) fast-spiking cell (FS), (**d**) burst-spiking cell (BS), (**e**) stuttering cell (ST), (**f**) irregular-spiking cell (IS). **g** Instantaneous spiking frequencies of ACC, REG, FS, and BS (*left*), ST (*middle*), and IS (*right*) plotted against the spike number in a train. **h** Percentage of interneurons in the BLA (left) and comparing the LA and BA (right) with respective firing pattern. **i** Percentage of interneurons with depolarizing sag potential in response to a hyperpolarizing current injection.

Whole-cell current-clamp recordings were obtained from GFP-positive neurons in the BA and LA of GAD67-GFP transgenic mice^20^. No differences were found in the passive membrane properties of interneurons in the two nuclei, and the overall resting membrane potential was −62.3 ± 0.6 mV (*n* = 63) with an input resistance of 252 ± 17 MΩ (*n* = 63). As previously described^16,21^, based on the pattern of action potential discharge evoked by somatic current injection, cells could be grouped into 6 subtypes: accommodating cells (ACC; Fig. 1a, g), regular-spiking cells (REG; Fig. 1b, g), fast-spiking cells (FS; Fig. 1c, g), burst-spiking cells (BS; Fig. 1d, g), stuttering cells (ST; Fig. 1e, g), and irregular-spiking cells (IS; Fig. 1f, g). Dividing these subtypes by nuclei, a higher proportion of fast and burst spikers were detected in the BA (Supplementary Table 1; Fig. 1h), consistent with its larger fraction of parvalbumin expressing neurons (Supplementary Fig. 1)^22,23^. Each type demonstrated a different proportion of cells that exhibited depolarizing sag in the voltage response to hyperpolarizing current injection (Supplementary Table 1; Fig. 1i) suggesting that the presence of hyperpolarization activated cation conductance (*I*_h_) is subtype specific.

### Interneurons receive thalamic and cortical auditory input

The BLA receives two streams of auditory input: one directly from the AT, and a more processed stream from the AC^24^. The AT is comprised of three nuclei: the medial geniculate body (MGN), which in turn is divided into the ventral (MGv), dorsal (MGd), and medial (MGm) nuclei; the suprageniculate nucleus (SG) and posterior intralaminar nucleus (PIN) of the posterior thalamus. All nuclei of the AT receive input from the inferior colliculus and project to the AC^25,26^, while only MGm, SG, PIN, and to a lesser extent the MGd, send direct projections to the BLA^27–29^. The AC, comprised of the primary AT (Te1) and neighboring temporal association cortex (TeA), has no direct projections from the inferior colliculus, but receives auditory innervation indirectly via the AT. Te1 is predominantly innervated by MGv^30–32^ but has sparse projections to the BLA^29,33^. In contrast, TeA which gets direct input from MGm, PIN, SG, and MGd also receives processed auditory input from Te1^28,29^ and sends long-range projections via the external capsule to densely innervate the BLA^26,29,33^. Thus, the BLA receives rapid auditory input directly from the thalamus, and a more processed but delayed input from the temporal association cortex, TeA^28,29^.

**Fig. 2:**
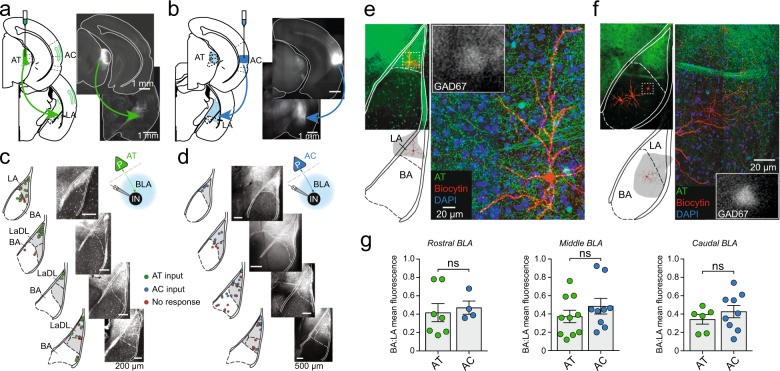
Auditory input targets the lateral amygdala (LA). **a**, **b** Virus encoding ChR2-eYFP was stereotactically targeted to the auditory thalamus (AT) **a** or the auditory cortex (AC) **b**. ChR2-eYFP expressing afferents from the AT (**c**) and AC (**d**) were detected throughout the rostro-caudal extent of the BLA (*right*). Schematics (*left*) show corresponding locations of interneurons that that received AT input (green), AC input (blue) or no input (red). **e**, **f** Shown are representative cells in the LA (**e**) and BA (**f**) recovered following immunohistochemistry for biocytin. AT afferents (CHr2-eYFP) are also shown (green) highlighting the higher density of auditory projections within the LA (**e**) compared to the BA (**f**). Insets show respective somatic GAD67-eGFP fluorescence for GABAergic identification. **g** Within-slice ratio of BA:LA fluorescence across the rostro-caudal range (*rostral BLA*, AT = 0.42 ± 0.1 (*n* = 7), AC = 0.47 ± 0.07 (*n* = 4), *p* = 0.6695; *middle BLA*, AT = 0.37 ± 0.07 (*n* = 10), AC = 0.48 ± 0.08 (*n* = 9), *p* = 0.3219; *caudal BLA*, AT = 0.34 ± 0.05 (*n* = 6), AC = 0.43 ± 0.7 (*n* = 9), *p* = 0.3403). Mean ± SEM (unpaired two-tailed *t* test with Welch’s correction).

**Fig. 3:**
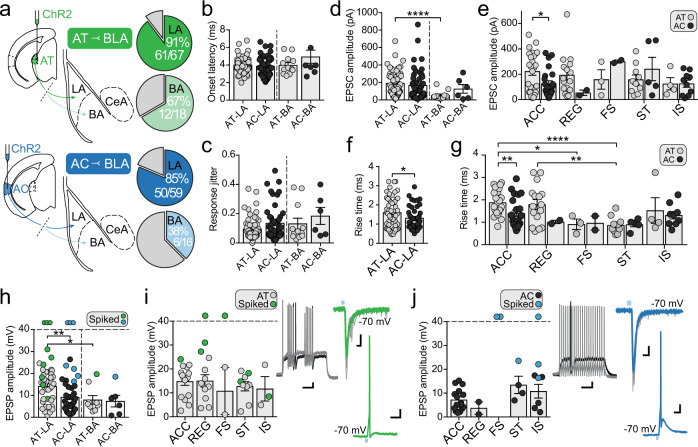
Auditory afferents from both the thalamus (AT) and cortex (AC) innervate all interneuron subtypes of the LA. **a** Schematics illustrating injection sites for AT (*upper*) and AC (*lower*) and recording sites in LA and BA. Inset: pie charts showing number of light-responsive interneurons in LA interneurons (dark green, dark blue) and BA (light green, light blue). **b** Onset latency of EPSCs in all cell types. **c** Response jitter in all cell types showing the variance in onset latency for light-evoked EPSCs. **d** Peak EPSC amplitude of light-evoked AT and AC inputs onto LA and BA interneurons (mean amplitude: AT LA EPSC = 191.2 ± 18.4 pA; AT BA EPSC = 52.6 ± 14.3 pA; *p* < 0.0001). **e** Amplitude of light-evoked EPSCs observed for each type of interneuron in the LA (AT ACC EPSC vs AC ACC EPSC, *p* = 0.0137). **f** Mean 10–90% rise time of light-evoked EPSC for AT and AC input onto LA interneurons (*p* = 0.0251). **g** EPSC rise times grouped by interneuron subtype (AT ACC vs AC ACC, *p* = 0.0093; AT ACC vs AT FS, *p* = 0.0325; AT ACC vs AT ST, *p* < 0.0001; AT REG vs AT ST, *p* = 0.0012. **h** Current-clamp recordings showing mean light-evoked EPSP amplitudes of AT and AC inputs to interneurons in the LA and BA (AT LA vs AC LA, *p* = 0.0038; AT LA vs AT BA, *p* = 0.0171). Neurons that reached threshold are marked by color, inputs that consistently drove suprathreshold responses are represented by a data point above the dashed line. **i**, **j** Peak EPSP amplitude evoked by AT (**i**) and AC (**j**) stimulation for each interneuron subtype in the LA. Neurons reaching threshold are marked as indicated. Inset: representative examples of suprathreshold light-evoked inputs (scale 10 mV, 20 ms) onto ST (AT) and FS (AC) IN subtypes (scale 10 mV, 100 ms), with corresponding synaptic currents voltage-clamped at −70 mV (scale 200 pA, 10 ms). Data = Mean ± SEM (unpaired two-tailed *t* test with Welch’s correction, *p < 0.05, **p < 0.01, ***p < 0.001, ****p < 0.0001).

To determine the nature of auditory input to BLA interneurons, light-gated channelrhodopsin-2 (ChR2-eYFP) was expressed in the AT (Fig. 2a) or TeA (Fig. 2b) to label thalamic and cortical inputs respectively. Consistent with previous anatomical tracer studies, visualization of transduced YFP-tagged ChR2 terminals from either AT (Fig. 2c) or AC (Fig. 2d) revealed strong labeling within the LA (Fig. 2e), with comparatively sparse innervation in the BA (Fig. 2f), across the entire rostro-caudal extent of the BLA (Fig. 2g). Whole-cell voltage-clamp recordings were then obtained from interneurons in the BLA. Light-activation of AT afferents revealed direct input in ~91% (61/67) of LA interneurons (Fig. 3a), and the evoked excitatory postsynaptic current (EPSC; Vh = −70 mV) had a peak amplitude of 191 ± 18 pA. For AC afferents, 85% (50/59) of LA interneurons received input (Fig. 3a), and the evoked EPSC had a peak amplitude of 167 ± 25 pA. Although each input was tested independently, these high innervation rates suggest most interneurons receive both AT and AC input.

In the BA, 67% (12/18) of interneurons received AT input (Fig. 3a) and it was significantly smaller (peak amplitude: 53 ± 14 pA; *p* < 0.0001) as compared to interneurons in the LA. AC input to BA interneurons was also sparse with 38% of cells (6/16; Fig. 3b) being innervated, but input to individual cells (127 ± 50 pA) was not significantly different to that in the LA (*p* = 0.5888). For all interneurons, light-evoked EPSCs were time locked to the onset of light stimulation (Fig. 3b) with small synaptic jitter (Fig. 3c), consistent with direct, monosynaptic connections.

These results show that there are clear differences in auditory input to interneurons in the LA and BA. Input from both AT and AC is larger to interneurons in the LA with more cells receiving input, and the overall amplitude of thalamic input to LA interneurons is significantly larger than to those in the BA (Fig. 3d). For cortical input, while fewer BA interneurons were innervated, the absolute size of the input is similar in the LA and BA. It should be noted though that BA interneurons with larger AC inputs were close to the LA/BA boundary, which can be difficult to define clearly. When comparing LA interneurons based on their discharge properties (Fig. 1), all six subtypes received input from both the AT and AC (Supplementary Table 2; Fig. 3e). However, while the total numbers of some subtypes were small, AT input to ACC neurons was significantly larger than AC input (*p* = 0.0137; Fig. 3e).

Interestingly, when we compared AT and AC inputs to individual interneurons, the rise time of AT EPSCs was significantly slower than that evoked by AC inputs (AT-LA 10–90% rise time = 1.6 ± 0.09 ms, *n* = 59; AC-LA rise time = 1.3 ± 0.08 ms, *n* = 46; *p* = 0.0482; Fig. 3f), suggesting that cortical inputs to these interneurons may be distributed electrotonically closer to the soma. However, there was no difference in the ability of inputs to drive cells to threshold. In the LA, thalamic input drove suprathreshold responses in 21% of cells (9/44), and cortical input could drive 19% (7/38) to threshold (Fig. 3h–j). All six types of interneuron (Fig. 3i) could be driven to spike by AT stimulation, whereas suprathreshold responses to AC stimulation were only observed in FS, ST, and IS subtypes (Fig. 3j).

**Fig. 4:**
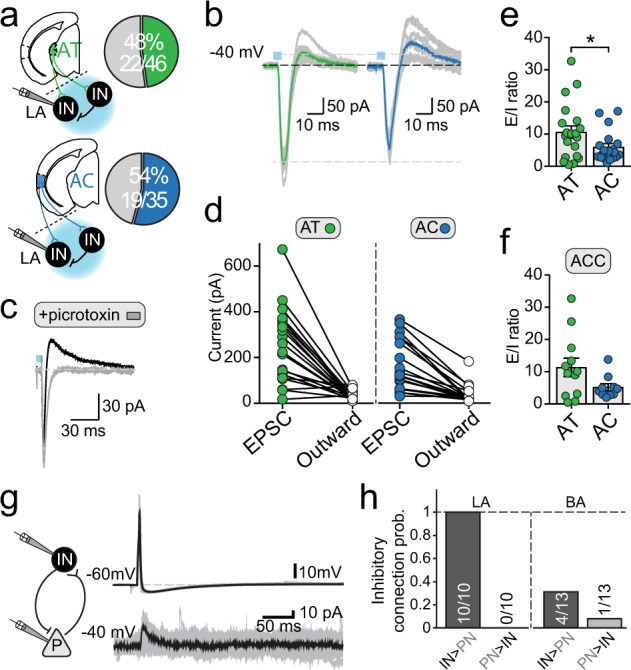
Auditory afferents evoked robust feed-forward inhibition. **a** Schematic illustrates assessment of light-evoked feed-forward inhibition by AT (*upper*) and AC (*lower*) afferents to interneurons in the LA. Pie charts show proportion of interneurons that had biphasic synaptic response to stimulation of AT (green) and AC (blue) input. These delayed outward currents (**b**) were sensitive to the GABA_A_ antagonist picrotoxin (**c**), indicating disynaptic inhibition. Quantification of the peak monosynaptic EPSC amplitude at −70 mV against the peak disynaptic IPSC amplitude at −40 mV (**d**) revealed a significantly smaller E/I ratio during light-evoked AC inputs compared to AT (AT LA IN E/I = 10.6 ± 1.9, *n* = 20; AC LA IN E/I = 5.9 ± 1.1, *n* = 19; *p* = 0.0453) (**e**), a trend similarly observed in LA IN ACC (*p* = 0.0637) (**f**). **g** Schematic of local feed-forward circuitry tested between interneurons (IN) and principal neurons (PN) within the LA or the BA. Inset: representative traces from a unidirectionally connected IN (*top*) and PN (*bottom*), where a somatically-evoked action potential in the presynaptic IN drove a time-locked outward current (held at −40 mV) in the postsynaptic PN. **h** Probability of connection between local IN and PN in the LA and BA.

Interneurons in the BLA make synaptic connections between cells of the same^21,34^ and different^10,34^ families. As most interneurons in the BLA receive auditory input and can drive these cells to threshold, we next tested if auditory inputs drove feed-forward inhibition on interneurons (Fig. 4a). With interneurons voltage clamped at a depolarized membrane potential (−40 mV), stimulation of either AT or AC afferents elicited biphasic synaptic responses (Fig. 4b) in approximately half the interneurons in the LA. The outward component of this biphasic response was blocked by GABA_A_ receptor antagonist picrotoxin (20 μM; Fig. 4c). To compare the level of feed-forward inhibition for the two inputs onto these cells, we calculated the excitation to inhibition (E/I) ratio. We found that AC input drove feed-forward inhibition with a smaller E/I ratio as compared to AT input (AT-LA E/I ratio = 10.6 ± 1.9; AC-LA E/I ratio = 5.9 ± 1.1; *p* = 0.0453), consistent with the stronger AT input. Though not significant, a similar difference was observed within the biphasic E/I ratio of AT and AC inputs on ACC interneurons (AT-ACC E/I ratio = 11.4 ± 2.8; AC-ACC E/I ratio = 5.2 ± 1.1; *p* = 0.0637; Fig. 4f). While we have not directly tested optically driven inhibition to principal cells, paired recordings revealed a high unidirectional inhibitory connection probability between interneurons and pyramidal cells in the LA (Fig. 4g, h), suggesting that auditory stimulation would likely drive feed-forward inhibition onto LA excitatory pyramidal neurons.

### LA and BA interneurons have divergent synaptic properties

**Fig. 5:**
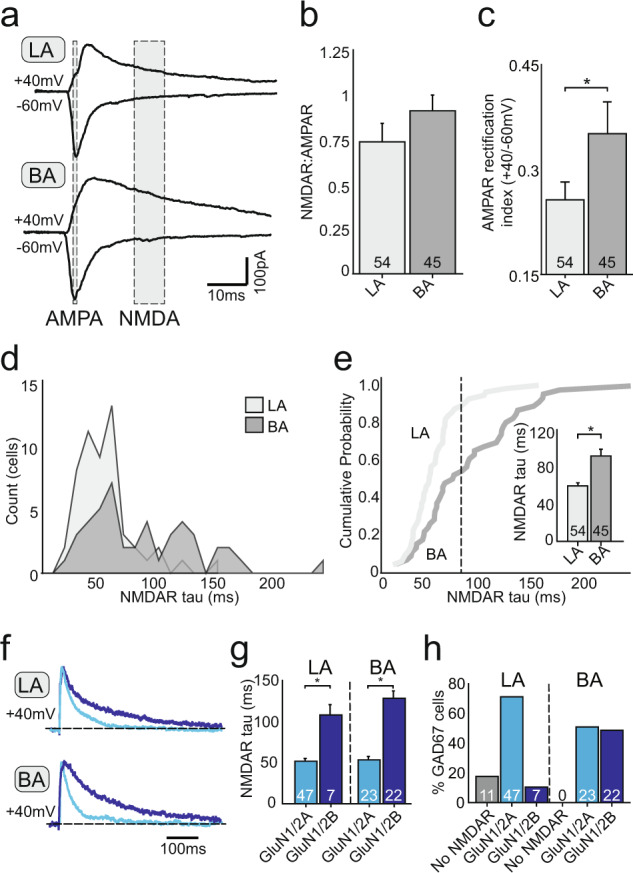
LA and BA IN populations have divergent glutamate receptor subunit compositions. **a** Representative EPSCs recorded from LA and BA IN in voltage-clamp at −60 mV and +40 mV. Shading shows regions for quantification of AMPA and NMDA receptor components. **b** NMDAR-to-AMPAR ratios for input to interneurons in the LA and BA. **c** AMPA receptors at synapses in the LA are more strongly rectifying (LA, 0.25 ± 0.02, *n* = 54; BA, 0.35 ± 0.04, *n* = 45; *p* = 0.015). **d** Distribution of NMDAR decay time constants for interneurons in the LA (light gray) and BA (dark gray). **e** Cumulative probability plot for NMDAR decay time constants in LA and BA. Inset: bar graph shows mean NMDAR decay time constant for LA and BA interneurons (*p* = 0.00018). **f** Representative traces (normalized to EPSC peak at +40 mV) for classes of NMDAR EPSCs recorded in LA and BA (light blue = GluN1/2A, dark blue = GluN1/2B). **g** Quantification of NMDAR decay time constant for GluN1/2A- or GluN1/2B-containing NMDAR in the LA and BA. **h** Proportion of interneurons in LA and BA categorized by NMDAR subunit stoichiometry (LA IN: 18% lack NMDAR, 72% contain GluN1/2A, 10% contain GluN1/2B; BA IN: 51% contain GluN1/2A, 49% contain GluN1/2B). Mean ± SEM (unpaired two-tailed *t* test with Welch’s correction, *p < 0.05).

Our results show clear asymmetries in auditory input to interneurons in the LA and BA. We have previously shown that in a population of interneurons in the LA, glutamatergic synapses express strongly rectifying AMPA receptors, and at these synapses there are few if any postsynaptic NMDA receptors^35^. We therefore compared the biophysical properties of glutamatergic synapses on interneurons in the LA and BA. Synaptic inputs were evoked using electrical stimulation of the internal or external capsules to evoke thalamic and cortical inputs, respectively^36,37^. It should be noted that while stimulation of the internal and external capsule has generally been accepted to recruit thalamic and cortical inputs respectively, other afferents such as those arising from the hippocampus, are also likely to be present. Electrical stimulation is indiscriminate as to the source of afferents stimulated, however, in these cells synaptic currents evoked by stimulation in the external or internal capsule, as well as those occurring spontaneously have identical kinetics, indicating that all glutamatergic synapses on individual interneurons express similar ionotropic receptors^38^. Using a cesium based internal solution, and with GABA_A_ receptors blocked, glutamatergic inputs were evoked at holding potentials of −60 mV and +40 mV, and the NMDA/AMPA receptor ratio was used as a measure of the relative synaptic weights of the stimulated input (see Methods). As reported previously^35^, a population of cells in the LA had synapses that lacked NMDA receptors (Fig. 5h). At synapses where NMDA receptors were present, the NMDA/AMPA ratio of inputs to interneurons was comparable between the LA and BA (BA, 0.90 ± 0.07, *n* = 45; LA, 0.73 ± 0.10, *n* = 54; *p* > 0.05; Fig. 5a, b; Supplementary Fig. 2a).

The AMPAR rectification index was measured as the ratio of peak AMPAR EPSC amplitude at +40 mV and −60 mV in the presence of NMDAR blocker d-AP5 (30 μM), and was significantly higher in BA interneurons (BA: 0.35 ± 0.04, *n* = 45; LA: 0.25 ± 0.02, *n* = 54; *p* < 0.05; Fig. 5a, c; Supplementary Fig. 2b), suggesting that glutamatergic synapses on BA interneurons contained fewer GluR2-lacking, calcium permeable (CP)-AMPA receptors.

We next assessed the subunit composition of NMDA receptor subunits by measuring the weighted decay time constant (τ) of the synaptic current at +40 mV in the presence of AMPAR blocker NBQX (10 μM)^39^. The distribution of time constants showed differences between interneurons in the LA and BA (Fig. 5d), which was also reflected in the cumulative probability plots (Fig. 5e). Overall, the NMDAR EPSC on interneurons in the LA had a faster decay time constant (59 ± 3 ms, *n* = 54) as compared to that in the BA (91 ± 7 ms, *n* = 45) (Fig. 5e inset; *p* = 0.001, K-S test), suggesting the presence of a higher percentage of GluN2A-containing heterodimeric NMDA receptors in LA interneurons^38–40^. To evaluate the proportion of interneurons expressing each type of NMDA receptor, LA and BA interneurons were separated into three classes based on the kinetics of NMDAR EPSCs: those expressing receptors largely containing GluN1/2A (τ < 80 ms), those expressing receptors containing GluN1/2B (τ > 80 ms), and those lacking NMDAR receptors (Fig. 5h)^38^. This analysis shows that in the LA, 18% of interneurons had synapses that did not express synaptic NMDARs (*n* = 11/64), 72% expressed NMDARs largely composed of GluN2A-heterodimers (*n* = 47/64), and 10% expressed GluN2B-containing NMDARs (*n* = 7/64). In contrast, in the BA, 51% of cells had synapses expressing mostly GluN2A-heterodimeric NMDARs (*n* = 23/45), 49% expressed GluN2B-containing NMDARs (*n* = 22/45), and no cells were found with synapses that lacked NMDA receptors (Fig. 5h).

### Interneuron LTP is restricted to the lateral amygdala

**Fig. 6:**
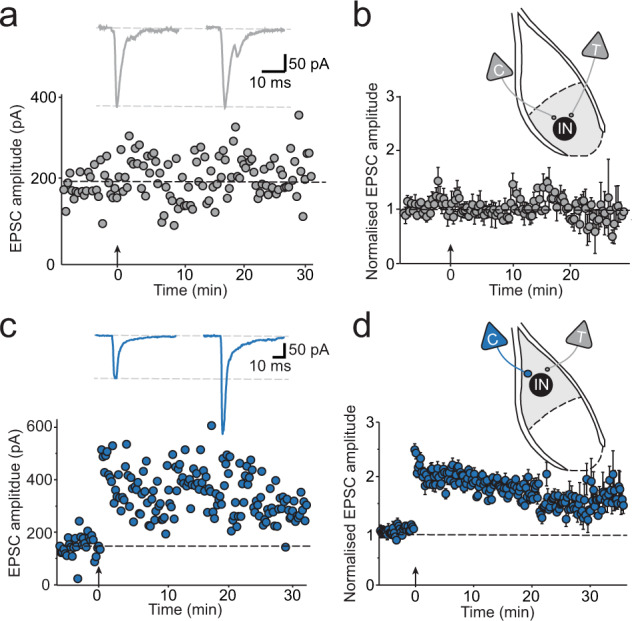
Interneurons in the BA are resilient to LTP induction. **a** EPSC amplitude evoked by cortical input stimulation plotted over time for a representative BA interneuron voltage-clamped at −60 mV. Tetanic stimulation was delivered at time zero, indicated by the arrow, and had no long-term impact on EPSC amplitude. Inset: average traces of baseline and post-tetanic EPSCs. **b** Averaged time course for LTP induction in BA IN (*n* = 8). (**c**) EPSC amplitude evoked by cortical input stimulation plotted over time for a representative LA interneuron voltage-clamped at −60 mV. Tetanic stimulation was delivered at time zero, indicated by the arrow, and shows immediate potentiation of the EPSC. Inset: average traces of baseline and post-tetanic EPSCs. **d** Average time course for LTP induction in LA IN (*n* = 12).

We have shown that excitatory inputs to interneurons in the BLA form synapses that largely contain GluR2-lacking AMPA receptors, but there is diversity in the types of NMDA receptors present. In the LA, cortical input to interneurons shows a form of NMDA receptor independent LTP, that is initiated by calcium influx via Ca^2+^ permeable (CP) GluR2-lacking AMPAR-receptors^35^ but is limited to cells that do not express GluN2B subunits^38^. Given the differences in auditory innervation and synaptic properties between the LA and the BA, we next asked if inputs to interneurons in the BA undergo LTP. All interneurons in the BA express synaptic NMDA receptors (Fig. 5h), however, tetanic stimulation of cortical input to interneurons in the BA failed to evoke LTP (*n* = 8; Fig. 6 a, b). In contrast, as reported previously^38^, tetanic stimulation of cortical inputs onto LA interneurons expressing GluN2A-containing NMDA receptors reliably evoked LTP (normalized EPSC amplitude = 1.5 ± 0.1; *n* = 9; Fig. 6c, d).

## Discussion

The BLA is a cortical-like structure that plays a central role in processing, storage, and retrieval of associative fear memories. It contains two main types of neuron: glutamatergic pyramidal-like neurons comprising ~85% of the population, while the remaining ~15% are GABAergic interneurons^4,41^. Interneurons in the BLA tightly control the excitability of principal neurons^13,42–45^ and play a key role in associative learning^10–13^. The BLA is anatomically divided into the LA and BA^46–48^, but functional studies treat interneurons of these nuclei as one population. In this study we have characterized interneurons in the LA and BA, along with their innervation by auditory inputs, and show that while sharing some properties, they also have clear differences.

Interneurons are a heterogenous population that are separated into groups by expression of cytosolic markers, electrical discharge properties, and the synaptic connections they make^16,17,49,50^. Of these, the best understood are those expressing PV and those expressing SOM. While these markers define two distinct developmental classes, there are different cell types within each. For example, among PV interneurons, some innervate the soma and axon initial segment, a different type innervates the soma alone, while another type innervates the proximal dendritic tree^16,21,22^, and they each have different physiological impacts^45,51^. Markers that separate these classes are not currently available, and we separated BLA interneurons on their discharge properties. Based on these criteria, cells were divided into six types: accommodating cells (ACC), regular-spiking cells (REG), fast-spiking cells (FS), stuttering cells (ST), irregular-spiking cells (IS), and burst-spiking cells (BS). All six classes have been previously described in the cerebral cortex^52^, and four types (ACC, FS, ST, IS) have been previously described in the BA^21,22^.

Our data show that the distribution of inhibitory interneurons in the BLA is different from other cortical and hippocampal regions. Whereas FS cells form ~30–50% of the interneuron population in the cortex and hippocampus, using a pan interneuron GFP-expressing mouse line (GAD67-EGFP), we find these neurons form a relatively minor population in the BLA. Interneurons in the BLA were initially distinguished by expression of calcium binding proteins, aspiny dendritic trees^16,41,48,53^, and electrophysiologically as cells with fast-spiking discharge properties. However, with the introduction of mice with genetically labeled interneurons, it is clear that not all interneurons in the BLA are FS cells^21,22^. Indeed, as shown here, the most common BLA interneuron subtype is accommodating, a firing pattern characteristic of BLA excitatory principal neurons^54,55^. Moreover, while fast-spiking is widely used to identify parvalbumin interneurons in the hippocampus and the cortex, we have shown that FS cells are only a proportion of the parvalbumin cell population in the BLA^21^. These electrophysiological data are complemented by our immunohistochemistry (Supplementary Fig. 1) the finding that only ~15% of cells in the BLA contained mRNA for parvalbumin and of these cells, only 25% (~4% of the total) were FS cells^53^.

Using channelrhodopsin to target thalamic and cortical auditory nuclei we find that auditory input targets all types of interneurons. Innervation of interneurons in the LA was stronger with more cells receiving input and of larger amplitude. Out data suggest that most interneurons receive both thalamic and cortical auditory input. About 20% of cells were driven to threshold, and strong disynaptic inhibitory responses were evoked in nearly half of all interneurons sampled. Both AT and AC afferents can drive spiking in FS and ST interneurons in the LA, firing patterns highly correlated with parvalbumin expression^21,22^. This pattern of connectivity with most cells receiving auditory input, coupled with robust disynaptic inhibition, is consistent with in vivo single unit recordings that show most PV interneurons are excited by an auditory CS but a large fraction are inhibited, while most SOM interneurons are inhibited, leading to disinhibition of local principal neurons^10^. By comparison, auditory projections to the BA innervated fewer interneurons, and evoked smaller synaptic inputs. This nucleus-specific difference is consistent with the finding that LA but not BA PV+ interneurons receive excitatory input from the AT, and that PV interneurons drove feed-forward inhibition in the LA but not the BA^56^. Together, these data show that auditory processing in the LA is tightly controlled by inhibitory networks that operate by a combination of feed-forward inhibition and disinhibitory control^18,56^. The clear differences in innervation patterns in the BLA also suggest differing roles for LA and BA interneurons during auditory fear conditioning.

As expected by the low expression of GluR2 subunits in interneurons^57^, AMPA receptors at glutamatergic inputs were inwardly rectifying^35,38^. This rectification was more prominent for interneurons in the LA as compared to the BA, suggesting a higher fraction of synaptic AMPA receptors lacking GluN2 subunits in LA interneurons. There were also clear differences in the distribution of synaptic NMDA receptors. Firstly, a small population of interneurons in the LA do not express synaptic NMDA receptors. These interneurons were initially described in the LA as FS interneurons^35,38^ and, consistent with the lower proportion of parvalbumin interneurons, they do not appear to be present in the BA. As FS interneurons are a proportionally small population, it is possible that we missed some of these cells in the BA. Of cells that express NMDA receptors, one population had receptors containing GluN2B subunits, while a different population expressed receptors that appear to be GluN2A only heterodimers^39,58^. This second population is proportionally larger in the LA, where it marks cells at which cortical inputs undergo long-term potentiation^38^. In the BA, however, expression of these two types of NMDA receptors was evenly distributed, and inputs to these cells do not appear to undergo LTP. The reason for this lack of LTP at inputs to interneurons in the BA is not clear. In the LA, LTP is seen at inputs that have steep inward rectification and is evoked by calcium influx via calcium permeable AMPA receptors^35,38^. As described above, synaptic AMPA receptors on interneurons in the BA show significantly less rectification, showing that the complement of GluR2-lacking receptors is lower. Thus, one possibility is that the calcium rise at synapses on BA interneurons is not sufficient to trigger LTP. We initially mapped auditory input to interneurons in the LA and BA using channelrhodopsin expressed in thalamic and cortical auditory nuclei. However, the biophysical analysis of glutamatergic input to these cells and whether they could undergo plasticity was done using extracellular stimulation in the internal and external capsules. Unlike optogenetic stimulation this technique will activate a range of inputs, raising the question of how these data can be reconciled with those obtained in the first part of our study. However, we have shown in the BLA that all glutamatergic synapses on particular interneurons show identical biophysical properties^38^, and thus thalamic and cortical auditory input to interneurons are not expected to be different, and consistent with this the only differences we detected were between interneurons in the LA and BA, rather than differences in input from the external and internal capsule. With regard to synaptic plasticity, as channelrhodopsin increases release probability, tetanic stimulation as a means of evoking plasticity is problematic. Our results again show that there are differences in plasticity between cells in the LA and BA rather than in the input. Thus, while the method of stimulation is different both optical stimulation and extracellular stimulation show that interneurons in the LA and BA should be treated as different populations.

What explains differences in the physiological properties of interneurons in the BLA? The BLA contains a population of precursor cells that gives rise to newborn interneurons in the adult that integrate into the local circuitry^59^. Newborn neurons will receive synaptic inputs that undergo a period of maturation, a process during which the biophysical properties and subunits of glutamatergic synapses change^60^. Thus, it is possible that some interneurons sampled in our study are still not fully mature, accounting for the differences in biophysical properties. The physiological role of NMDA receptors in BLA interneurons is not completely clear, we suggest that like cytosolic markers and transcription factors that are used as markers for developmental fate mapping^15^, expression of ionotropic glutamate receptors may also be developmental markers^61^ that delineate different interneuron types and mark specific circuits in the LA and BA.

The BLA plays a key role in auditory fear conditioning, and a host of studies have identified changes in the response of BLA pyramidal cells and interneurons to auditory input following fear learning^6,8^. However, not all studies disentangle recordings obtained from cells in the LA from those in the neighboring the BA. Moreover, using cytosolic markers such as PV and SOM, interneurons are generally treated as single populations while there is clearly significant diversity even within these populations. In part, this is no doubt due to the difficulty in separating these nuclei, and different cell types, particularly in vivo. Synaptic plasticity of input to the BLA is widely accepted to underpin fear conditioning. This plasticity has been thought to be of input to principal neurons in the BLA^8^. It is clear that interneurons play key roles in fear learning^6,10^, and following fear conditioning, the response of some interneurons to the CS also changes^62^ suggesting that synaptic plasticity of input to interneurons plays a role in fear learning. It will be interesting to identify the cell types that mediate these responses, and our data suggests that they are likely to be located in the LA.

## Methods

### Animals

All experimental and animal care procedures were in accordance with the Australian Code of Practice for the Care and Use of Animals for Scientific Purposes and approved by the University of Queensland Animal Ethics Committee. Predominantly GAD67-GFP knock-in mice on a C57BL/6 background were used, which allowed for the visual differentiation of GABAergic interneurons from excitatory neurons in acute slice recording conditions.

### Virus

Third generation lentiviruses were produced in house for the transgene Channelrhodopsin-2 [pLenti-synapsin-hChR2(H134R)-eYFP-WPRE, kind gift from Karl Deisseroth, Stanford University]. Adeno-associated viruses (AAV) were obtained from Penn Vector Core (AAV2/5-hSyn-hChR2(H134R)-eYFP-WPRE-hGh).

### Surgery

Mice (p21-60) were anaesthetized with a ketamine (100 mg/kg)/xylazine (20 mg/kg) mixture intraperitoneally, and the head shaved and secured in a stereotaxic frame. Rectal temperature was monitored and maintained at 37 ± 0.5 °C throughout the procedure by a feedback-controlled heat pad. The scalp was hemisected and secured laterally, and a small craniotomy was performed unilaterally using a dental drill, in accordance with stereotaxic coordinates previously devised for each target region (AC: AP −3.0, ML + 4.5, DV −3.0; AT: AP −3.0, ML + 2.0, DV −3.0; in mm relative to bregma). Viral solutions were delivered to the required depth using a glass pipette pulled to a long taper (~10 mm), tip-filled with virus via capillary action, and placed on ice while the injection site was prepared to maximize viral infectious unit (IU) titre. Injection pipettes were initially positioned 0.05 mm lower than the target coordinate for the period of 10 min, then retracted to the target depth in order to create a small trough for the virus to fill. 0.3 μL of AAV, or 2 μL of lentivirus, was pressure injected to the target coordinate (for both AT and AC) at a rate of 0.1 μL/1 min, which produced equivalent and sufficient tissue volume of somatic transgene expression. Final injection volumes were calculated from the decrease in meniscus height, which was monitored using a magnified monocular scope with transecting cross-hairs, attached to a Vernier scale. Pipettes were held in place for 10 min following virus injection, and then slowly retracted to avoid diffusion of the viral solution into the injection tract. While anaesthetized, subjects were removed from the stereotaxic frame, and the scalp wound was sealed using tissue adhesive (3 M Vetbond, n-butyl cyanoacrylate). A bolus dose of the antibiotic Baytril (5 mg/kg) and analgesic Torbugesic (2 mg/kg) were then administered subcutaneously, each separately diluted with 1 mL of sterile saline (9 g/L NaCl) for hydration. Subjects recovered on a heat pad at 37 ± 0.5 °C until awake and autonomous, and recovered for 4–8 weeks to ensure sufficient transgene expression for light-evoked synaptic terminal release.

### Electrophysiology

Following halothane or isoflurane anesthesia, animals were decapitated and the brain rapidly removed and submerged in ice-cold, oxygenated artificial cerebrospinal fluid (aCSF) containing (in mM): 87 NaCl, 2.5 KCl, 25 NaHCO_3_, 25 glucose, 50 sucrose, 4 MgCl_2_, 0.5 CaCl_2_, and 1.2 NaH_2_PO_4_. Coronal slices (300μm) containing the BLA and injection sites were prepared using a Vibratome (VT1000S, Leica) and incubated at 32 °C for 30 min in aCSF, containing (in mM): 118 NaCl, 2.5 KCl, 25 NaHCO_3_, 10 glucose, 1.3 MgCl_2_, 2.5 CaCl_2_, and 1.2 NaH_2_PO_4_. Slices were then equilibrated to room temperature for at least 30 min before being transferred to the recording chamber. During recordings, slices were perfused with oxygenated aCSF, heated to 32 ± 2 °C and secured with a platinum harp strung with parallel nylon threads. Recording pipettes were fabricated from borosilicate glass and pulled to a tip resistance between 3 and 5 MΩ (GC150F, 1.5 mm, Harvard Apparatus, UK) when filled with internal solution containing (in mM): 135 KMeSO_4_, 8 NaCl, 10 HEPES, 2 Mg_2_ATP, 0.3 Na_3_GTP, 0.1 spermine, 7 phosphocreatine, 8 biocytin, and 0.3 EGTA. Some recordings were performed with a cesium based internal, which contained (in mM): 135 CsMeSO_4_, 8 NaCl, 10 HEPES, 2 Mg_2_ATP, 0.3 Na_3_GTP, 0.1 spermine, 7 phosphocreatine, 8 biocytin, and 0.3 EGTA. GABA_A_ antagonist picrotoxin (100 µM) was added to the ACSF where specified. Whole-cell patch-clamp recordings were made from eGFP expressing neurons in the LA and BA visualized using an upright microscope (Olympus BX50WI, Olympus Optical, Tokyo, Japan) equipped with a fluorescence attachment (100 W Olympus mercury burner). The boundary between the LA and the BA was demarcated relative to the location of clusters of GAD67-eGFP-positive GABAergic intercalated cells that border the BLA within the internal and external capsules. For optogenetics recordings, the position of each soma was imaged using a ×5 objective under bright field illumination, as approximated by the tip of the recording electrode, using iMovie software and an external analog/digital hardware converter (Canopus, ADVC55), which was then overlaid offline with the mouse brain atlas to confirm LA or BA categorization. Where ChR2-positive axon density and fluorescence intensity was too bright to visualize GAD67-positive interneuron somas, spontaneous EPSC decay time constants were used to differentiate interneurons (<4 ms) from principal neurons (>4 ms)^35,63^. Current-clamp and voltage-clamp recordings were made using a MultiClamp 700B amplifier (Molecular Devices). Recordings were filtered at 6 kHz, and digitized at 10–20 kHz using an ITC-18 board (InstruTech, Port Washington, NY) attached to an iMac. Recordings were acquired and analyzed offline using Axograph software for Windows (Axograph X, version 1.4.4), run through virtual machine software (VMWare Fusion 6).

To estimate the intrinsic membrane properties of interneurons, somatic current injections were applied in 500 ms square pulses at 50 pA incremental steps from −100 to 600 pA. Synaptic inputs were evoked at 0.1 Hz electrically (Digitimer, DS2A) or optically using 5 ms pulses of whole-field illumination at blue excitation wavelengths ~470 nm (Cairn OptoLED), and recorded in whole-cell patch-clamp configuration. Light-gated synaptic inputs met monosynaptic inclusion criteria when onset latency was <7 ms, synaptic jitter was <0.5, and average input amplitude was >5 pA (cut-off threshold for a miniature EPSC event). To examine synaptic properties, EPSCs were recorded in voltage-clamp at holding potentials of −60 and +40 mV, to determine: (1) NMDAR-to-AMPAR ratio; (2) AMPAR rectification in the presence of the NMDAR blocker AP5 (30 μM); (3) kinetics of the NMDAR mediated EPSC in the presence of the AMPAR blocker 2,3-Dihydroxy-6-nitro-7-sulfamoyl-benzo(f)quinoxaline-2,3-dion (NBQX, 10 μM). Bipolar stimulating electrodes were placed on the external capsule (EC) to stimulate cortical inputs, and medially within the internal capsule (IC) to stimulate thalamic inputs. The NMDAR/AMPAR ratio was calculated as the NMDA current at 25 ms after the peak of the dual component EPSC at +40 mV, divided by the peak AMPAR-mediated EPSC at −60 mV. The rectification index of AMPAR-mediated EPSCs was calculated as the peak amplitude at +40 mV divided by the peak amplitude at −60 mV. For comparison of NMDAR EPSC deactivation kinetics, 5–10 electrically evoked EPSCs were recorded at +40 mV and averaged. The synaptic current decays were fitted with a double exponential equation of the form: I(t) = *I*_f_exp(−*t*/τ_f_) + *I*_s_exp(−*t*/τ_s_), where *I*_f_ and *I*_s_ are the amplitudes of the fast and slow decay components, and τ_f_ and τ_s_ are their respective decay time constants. The weighted time constant was calculated as: τ_W_ = (*I*_f_/(*I*_f_ + *I*_s_))τ_f_ + (*I*_s_/(*I*_f_ + *I*_s_))τ_s_, and was used for statistical comparisons of the decay times of recorded EPSCs. LTP was evoked with a tetanic stimulus (100 Hz, 300 ms × 3, 10 s interval).

### Immunohistochemistry

For biocytin recovery and immunohistochemistry, brain slices were fixed in 4% paraformaldehyde in 0.1 M PBS overnight at 4 °C. Slices were washed three times with 0.1 M PBS, and then blocked and permeabilized with a blocking solution (PBS 0.1 M, bovine serum albumin 3%, saponin 0.1%, and sodium azide 0.05%) for 30 min at room temperature. Slices were then washed three times in 0.1 M PBS and incubated in blocking solution containing anti-GFP primary antibodies that recognize eYFP (anti-GFP-mouse 1:2000, Millipore; or anti-GFP-chicken 1:4000, Aves) for 2 days at room temperature on an orbital shaker. Slices were then washed three times for 15 min each time in 0.1 M PBS, then incubated in blocking solution containing Alexa Fluor-conjugated species-specific secondary antibodies (anti-mouse-AF488 1:1000, Molecular Probes; anti-chicken-AF488 at 1:1000, Molecular Probes) and streptavidin (Alexa Fluor 555 at 1:1000, Invitrogen) for 2 h at room temperature on an orbital shaker. Slices were then washed three times in 0.1 M PBS, briefly rinsed in saline (9 g/L NaCl) containing DAPI, then mounted from 0.1 M PBS into fluorescent mounting media (DakoCyomation) onto glass slides and coverslipped. Slices were imaged using an upright Axio Imager (Zeiss) microscope (5x or 20x objective), equipped with Zen Software (Zeiss), and an ApoTome grid for optical fluorescence sectioning. Images were produced by flattening z-stacks to a maximum projection image using the Z Project function within Fiji (ImageJ, 1.47 g). Within-slice BA:LA mean fluorescence ratios were quantified using Fiji, with LA and BA ROI boundaries determined using the mouse brain atlas. Ranges across the rostro-caudal axis were defined as (in mm relative to bregma): *rostral BLA* −0.94 to −1.22, *middle BLA* −1.46 to −1.7, *caudal BLA* −1.82 to −2.06. For immunohistochemical characterization of GABAergic interneuron subtypes, GAD67-GFP-positive mice were anaesthetized by an intraparietal injection of 1 ml/kg pentobarbitone and perfused with 4% paraformaldehyde-PBS. Extracted brains were stored overnight in the perfusion solution at 4 °C. Coronal sections (50 μm) containing the amygdala were serially washed 4 times in PBS, then blocked using a blocking buffer (PBS + 0.1% triton X-100 + 2% bovine serum albumin + 2% goat serum) for 30 min. The following dilutions of primary antibodies were used: 1:1000 anti-parvalbumin (Sigma-Aldrich), 1:1000 anti-calbindin (Sigma-Aldrich), 1:200 anti-somatostatin (Sigma-Aldrich), and 1:1000 anti-calretinin (Sigma-Aldrich). Primary antibodies were made up separately in incubation buffers (0.1% goat serum+ 0.4% Triton X-100), and sections were incubated with antibodies in a dark room at 4 °C overnight. After 4 × 10 min washes in PBS, sections were transferred to blocking buffer containing the corresponding species-specific fluorescently-tagged secondary antibody for incubation at room temperature for 2 h. The sections were then washed, mounted, coverslipped and imaged using a fluorescence microscope (Zeiss Axioplan 2). Counts of fluorescent cells were performed by eye using Adobe Photoshop, and the percentage of colocalisation was normalized to the total number of GAD67-GFP cells in the BLA.

### Reporting summary

Further information on research design is available in the Nature Research Reporting Summary linked to this article.

## Supplementary information

Supplementary Data

Reporting Summary

## Data Availability

All data are available in the main text or the Supplementary Materials.
