# Supplementary material for: Diversity of interneurons in the lateral and basal amygdala
Source: NPJ Sci Learn. 2020 Aug 3;5:10. doi: 10.1038/s41539-020-0071-z (PMC7400739; doi:10.1038/s41539-020-0071-z)

**Supplementary material**

# Diversity of interneurons in the lateral and basal amygdala

Jai S. Polepalli[1,2, 4], Helen Gooch[1,4] and Pankaj Sah[1,3*]

[1] Queensland Brain Institute, University of Queensland, St Lucia, QLD, 4072, Australia

[2] Department of Anatomy, Yong Loo Lin School of Medicine, National University of Singapore, Singapore.

[3] Brain Research Centre and Department of Biology, Southern University of Science and Technology, Nanshan District, Shenzhen, Guangdong Province, P. R. China

[4] These authors contributed equally to this work.

**Supplementary Table 1. Active and passive properties of BLA GAD67-positive interneuron subtypes.** (ACC, accommodating cells; REG, regular-spiking cells; FS, fast-spiking cells; BS, burst-spiking cells; ST, stuttering cells; IS, irregular-spiking cells; RMP, resting membrane potential; $R_{input}$, input resistance; AP, action potential; AHP, afterhyperpolarization).

| IN | RMP (mV) | Ri (MΩ) | AP ½-width (ms) | AHP amp (mV) | Proportion (BLA) | Proportion sag | Proportion (LA) | Proportion (BA) |
|---|---|---|---|---|---|---|---|---|
| ACC | -62.2 ± 1.1 | 245 ± 29 | 1.03 ± 0.08 | -24.3 ± 1.8 | 21/63 (33%) | 12/21 (57%) | 63/145 (44%) | 18/68 (27%) |
| REG | -59.3 ± 1.1 | 300 ± 38 | 0.75 ± 0.09 | -29.9 ± 2.5 | 9/63 (14%) | 2/9 (22%) | 25/145 (17%) | 16/68 (24%) |
| FS | -59.0 ± 2.1 | 289 ± 59 | 0.56 ± 0.04 | -33.8 ± 5.9 | 3/63 (5%) | 0/3 | 5/145 (3%) | 4/68 (6%) |
| BS | -63.6 ± 2.4 | 267 ± 39 | 0.56 ± 0.03 | -34.6 ± 4.3 | 4/63 (8%) | 4/4 (100%) | 8/145 (6%) | 8/68 (12%) |
| ST | -64.4 ± 0.1 | 243 ± 36 | 0.8 ± 0.05 | -25.8 ± 1.3 | 16/63 (25%) | 12/16 (75%) | 25/145 (17%) | 12/68 (18%) |
| IS | -63.8 ± 1.4 | 220 ± 34 | 0.85 ± 0.10 | -26.6 ± 1.0 | 10/63 (16%) | 6/10 (60%) | 19/145 (13%) | 10/68 (15%) |
| | | | | **TOTAL** | **63/63** | **36/63** | **145/213** | **68/213** |

**Supplementary Table 2. AT and AC input properties for LA interneuron subgroups.** (ACC, accommodating cells; REG, regular-spiking cells; FS, fast-spiking cells; BS, burst-spiking cells; ST, stuttering cells; IS, irregular-spiking cells; EPSC, excitatory postsynaptic current; EPSP, excitatory postsynaptic potential).

| | **EPSC amplitude (pA)** | | **EPSC rise time (ms)** | | **EPSP amplitude (mV)** | |
|---|---|---|---|---|---|---|
| **IN subtype** | **AT** | **AC** | **AT** | **AC** | **AT** | **AC** |
| ACC | 222.8 ± 30 (n = 26/26) | 126 ± 23 * (n = 23/27) | 1.88 ± 0.11 (n = 24) | 1.4 ± 0.14 ** (n = 23) | 14.8 ± 1.6 (n = 16) | 7.2 ± 1.1 (n = 16) |
| REG | 194.4 ± 40 (n = 16/18) | 54.7 ± 21 (n = 2/3) | 1.79 ± 0.22 (n = 16) | 1.02 ± 0.07 (n = 2) | 15.6 ± 2.8 (n = 11) | 3.7 ± 2.6 (n = 2) |
| FS | 159.2 ± 77 (n = 3/3) | 300.8 ± 12 (n = 2/2) | 0.91 ± 0.23 (n = 3) | 0.97 ± 0.31 (n = 2) | 10.8 ± 9.9 (n = 2) | Suprathreshold |
| BS | 110.3 (n = 1/3) | 680.8 (n = 1/3) | 1.7 (n = 1) | 2.03 (n = 1) | Suprathreshold (n = 1) | 18.9 (n = 1) |
| ST | 158.1 ± 36 (n = 10/10) | 241.7 ± 93 (n = 5/5) | 0.87 ± 0.12 (n = 10) | 0.91 ± 0.11 (n = 5) | 12.8 ± 1.9 (n = 9) | 13.5 ± 3.6 (n = 4) |
| IS | 125 ± 49 (n = 4/5) | 126 ± 28 (n = 10/11) | 1.53 ± 0.57 (n = 4) | 1.31 ± 0.16 (n = 9) | 11.7 ± 5.1 (n = 3) | 12 ± 3.0 (n = 7) |

**Supplementary Figure 1. Quantification of immunohistochemical interneuron markers for GABAergic interneurons in the mouse BLA.**
**(a-d)** BLA immunoreactivity for parvalbumin (**a**), calretinin (**b**), calbindin (**c**), and somatostatin (**d**), colocalised with GFP (*top*) from a *GAD67-GFP* transgenic mouse. Dashed lines indicate the boundary between lateral (LA) and basal (BA) amygdala.

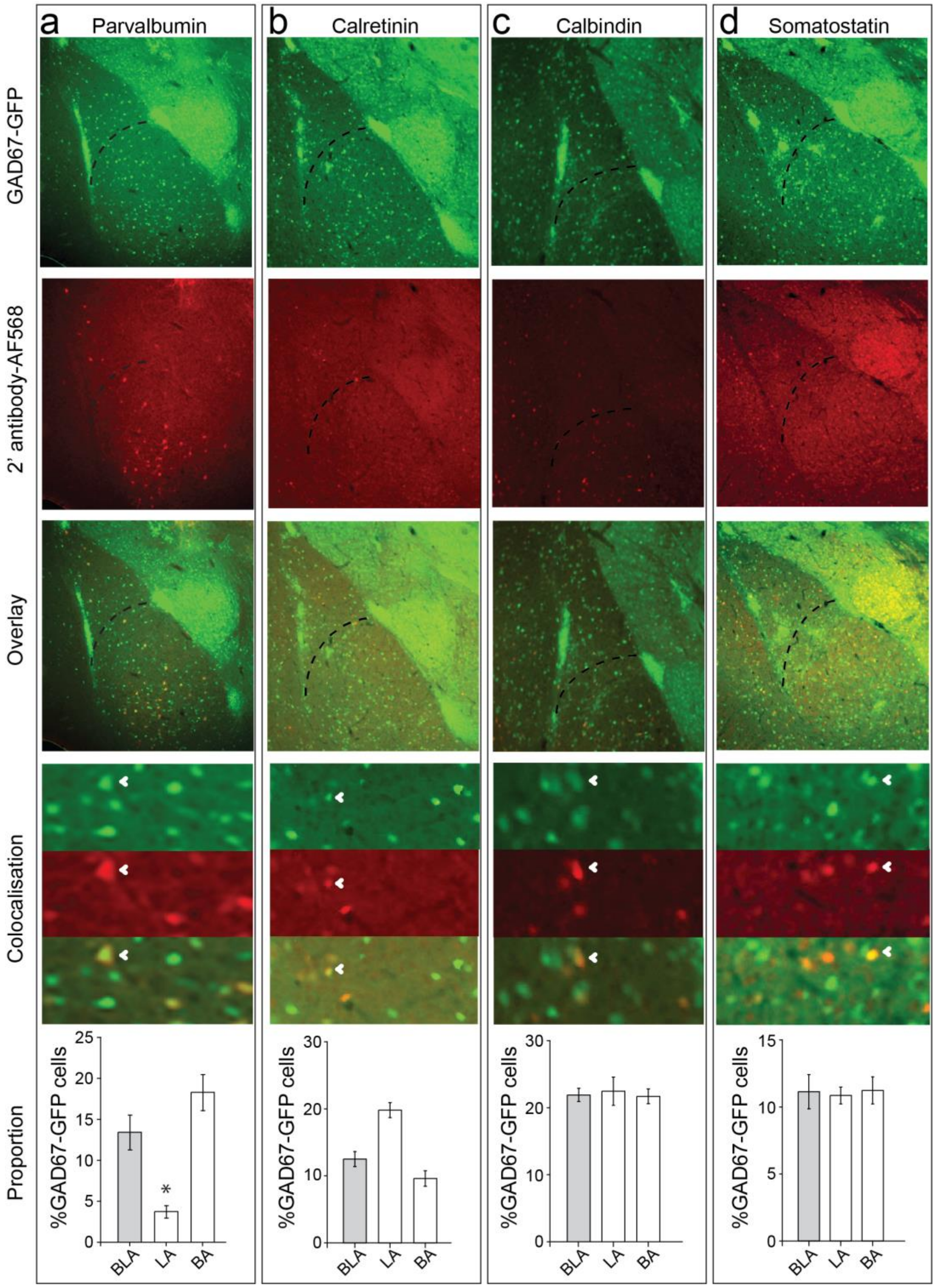

**Supplementary Figure 2. Thalamic and cortical inputs onto LA interneurons have similar synaptic properties.**

**(a)** NMDAR-to-AMPAR ratios of thalamic and cortical inputs onto LA INs. *Inset*, representative traces of AMPA-R and NMDA-R EPSCs recorded in voltage clamp at -60mV and +40mV, respectively. **(b)** AMPA-R rectification index of thalamic and cortical inputs onto LA INs. *Inset*, representative traces of AMPA-R EPSC recorded in voltage clamp at -60mV and +40mV).

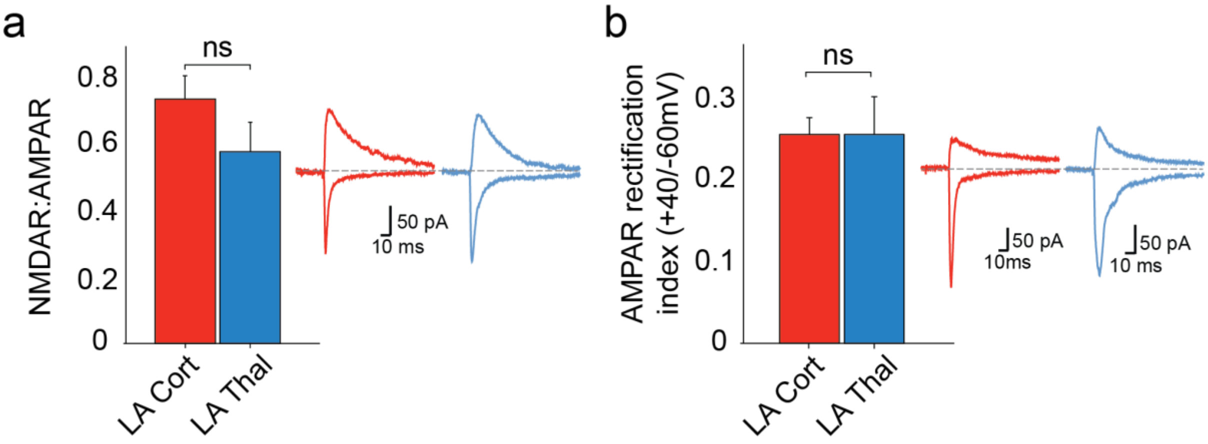

Supplement: Supplementary file 1 — Supplementary Data [file 41539_2020_71_MOESM1_ESM.pdf]
